# Mckenzie therapy plus simplified Wuqinxi exercise for improving lumbar spine motor function in chronic low back pain: a randomized controlled trial protocol

**DOI:** 10.3389/fspor.2026.1807993

**Published:** 2026-05-07

**Authors:** Fei Li, Yanjun Cao, Wei Feng

**Affiliations:** 1 Department of Rehabilitation Medicine, Tongji University Affiliated Shanghai Fourth People's Hospital, Shanghai, China; 2 School of Rehabilitation Science, Shanghai University of Traditional Chinese Medicine, Shanghai, China; 3 Shanghai Second Rehabilitation Hospital, Shanghai, China

**Keywords:** chronic low back, combined therapy, McKenzie therapy, traditional martial arts, Wuqinxis

## Abstract

**Background:**

Chronic low back discomfort may result in functional restrictions that can be incapacitating and cause disruptions to family life. McKenzie therapy (MT) can reduce pain by improving the functional structure of the lumbar spine through exercise and posture, while the simplified Wuqinxis (WQXs) can play a dual role in adjusting physical form and mental state as a traditional Chinese medicine exercise. This study aims to combine the concepts of the two therapies, to develop a more effective and easy exercise prescription for the prevention and treatment of lower back pain, and to explore the biomechanical effects and clinical efficacy.

**Methods and analysis:**

A randomized controlled trial will be conducted to compare the efficacy of WQXs with or without MT on patients with chronic low back pain. A total of 74 patients will be randomly assigned to an experimental group (MT and WQXs) and a control group (WQXs). Lumbar range of motion and health-related quality of life in patients with chronic low back pain will be assessed using the range of motion scale, visual analogue scale, and the SF-36 scale. Additionally, surface electromyography (sEMG) and isokinetic muscle strength testing will be employed to evaluate the mechanical properties of the core muscles in the lumbopelvic region. Consider a 20% dropout rate, 74 cases will be gathered in total, taking into account a 20% dropout rate.

**Results:**

to ascertain whether the exercise prescription combining the two therapies is effective for treating chronic low back pain.

**Clinical Trial Registration:**

Chinese Clinical Trial Registry, identifier ChiCTR2300075499.

## Strengths and limitations of this study

- This is a small-scale randomized controlled trial investigating the effects of active exercise combined with passive physical therapy on lumbar mobility, pain, and quality of life.
- This integrated approach, which combines Eastern and Western therapeutic modalities, may offer a novel paradigm for enhancing the rehabilitation of patients with chronic lower back pain.
- The outcomes will provide crucial insights related to lumbar spine functionality, encompassing aspects of pain, range of motion, and capabilities in daily living.
- This study is characterized by a modest sample size, necessitates further validation through the acquisition of additional data.

## 1 Background

Low back pain (LBP) constitutes a pervasive condition, exerting its impact on individuals spanning the entire globe (1), which includes three primary components: axial lumbosacral pain, radicular pain, and referred pain. Axial lumbosacral pain encompasses the lumbar region (L1-5 vertebral level) and sacral region (S1 to sacrococcygeal junction). Radicular pain arises from nerve or dorsal root ganglion distribution into the extremities along the dermatomal pathway, while metastatic pain refers to pain that emanates from a distant source along the non-cutaneous trajectory. All the three types of LBP are prevalent worldwide (2, 3) and can significantly impair patients’ quality of life (4).

LBP is also classified by symptom duration: acute (<6 weeks), subacute (6–12 weeks), or chronic (>12 weeks) (5, 6). Evidence suggests that approximately 20% of patients with acute low back pain progress to chronic back pain over time (7). In developed countries, treatment for chronic LBP costs at least one million dollars annually (8). This has resulted in high medical expenses and loss of productivity, posing significant socioeconomic challenges (9). As a result, understanding the underlying mechanisms, optimizing treatment strategies, and improving clinical outcomes for chronic LBP have become increasingly complex and critical priorities for healthcare professionals.

Treatment of chronic LBP is multifactorial, involving physicians, patients, families, and society. Chronic low back pain (CLBP) exhibits a high incidence among young adults and middle-aged individuals. As this age group constitutes the primary labor force, they demonstrate substantial rehabilitation needs (10). Approximately one-third of low back pain patients experience moderate discomfort. Patients tend to avoid opioid analgesics unless their pain is severe and fails to respond to more conservative medications (11). Psychosocial and emotional factors play a significant role in the prognosis and persistence of chronic LBP (12). The National Institute for Health and Care Excellence (NICE) (13) recommends early management and treatment of chronic lower back pain through information, education, patient preference, regular exercise, and physical activity. NICE also endorses Mckenzie therapy (MT) for individuals with LBP. Manual therapy entails neurophysiological interventions such as reducing inflammation, decreasing spinal excitability and pain sensitivity, altering the activity of cortical areas involved in pain processing, and stimulating the sympathetic nervous system (14, 15). While MT offers a personalized treatment approach for chronic LBP, its overall clinical efficacy remains a subject of debate (16, 17).

The WQXs was created by Hua Tuo, which is renowned as a famous doctor in Chinese history (18), This mind–body practice imitates the movements and breathing patterns of the five animals: tiger, bear, crane, monkey, and deer, and integrate breathing postures and movements, which can lessen stress on the injured ligaments of the lumbar spine, alleviate pain, and improve function (19–22). Furthermore, low-load lumbar multilevel isometric exercises, such as those embedded in WQXs, have been shown to enhance spinal control and stability (23–25). Both the McKenzie Method and Wuqinxi are applicable to patients with non-specific low back pain (NSLBP). Furthermore, both interventions possess a solid foundation of clinical application across different age groups. But the efficacy of the combination of WQXs and MT, an active movement combined with a passive rehabilitation therapy, is yet to be determined. This study aims to conduct a small randomized controlled trial to observe the impact of this combination on lumbar spine mobility, pain, and quality of life. A novel treatment program that integrates both approaches will be offered to patients.

## 2 Methods

### 2.1 Design

**Figure 1:**
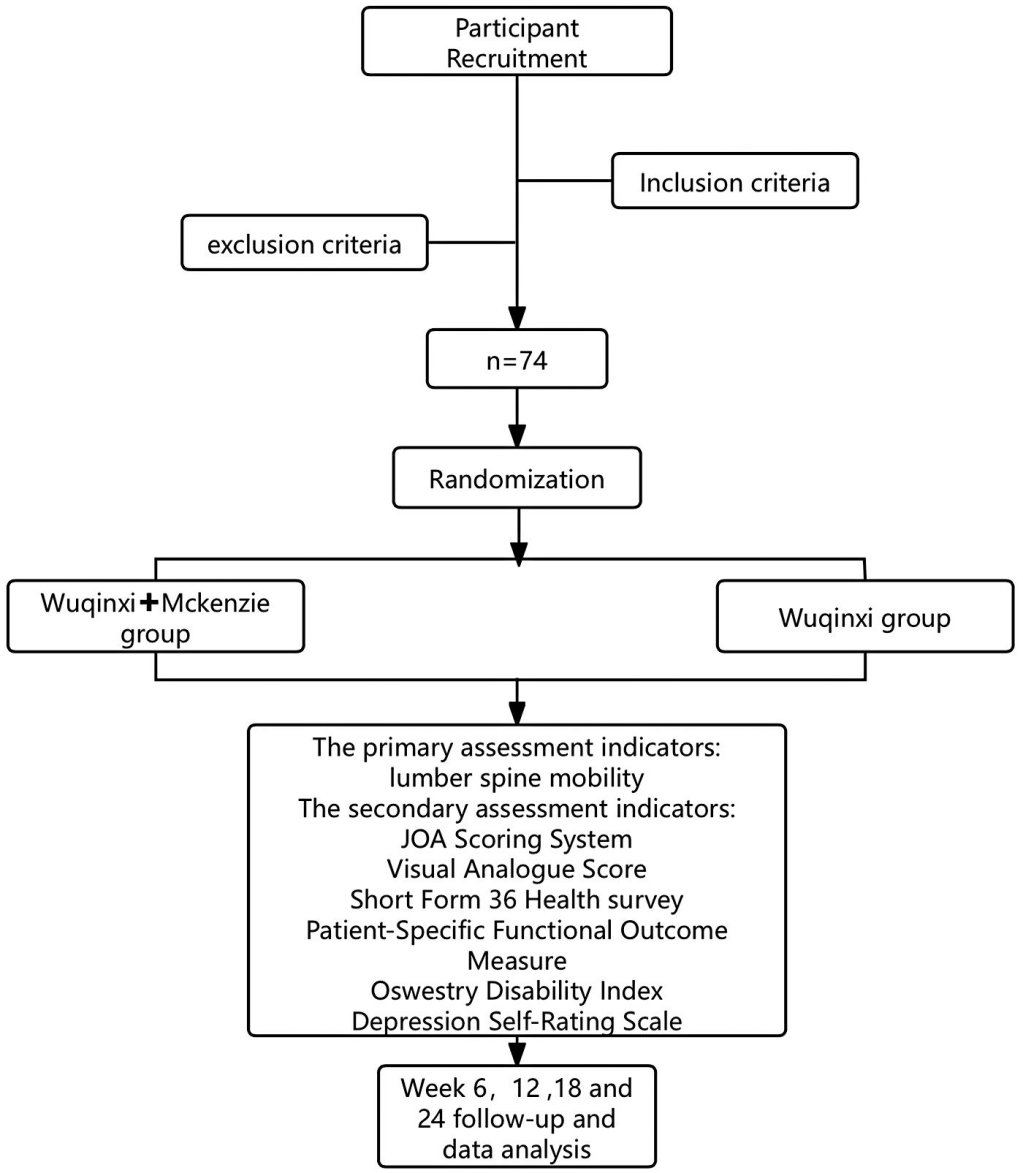
Aims and study flow diagram.

All patients are recruited from community residents in the Hongkou and Yangpu districts of Shanghai voluntarily. After obtaining informed consent, they are randomly assigned to either control group or experimental group: control group receive a simplified WQXs intervention, which is refer to as WG, and experimental group receive a combination of simplified WQXs and MT intervention, which is refer to as WMG. The study observation period was 24 weeks. The range of motion (ROM) of the lumbar spine in flexion, extension, and lateral flexion was measured using an electrogoniometer. Pain and the quality of life of patients with chronic low back pain is assessed using scales such as the visual analogue scale (VAS) and the SF-36 questionnaire. The mechanical properties of the lumbago core muscles are evaluated using surface electromyography and isokinetic muscle strength tests to explore the underlying biomechanical mechanisms and enhance clinical efficacy. The following covariates were collected at baseline: age, sex, body mass index (BMI), occupational type (sedentary/manual labor), daily physical activity level, disease duration, and history of low back pain. The impact of these factors on low back pain was analyzed (Figure 1).

**Eligibility criteria**

Inclusion criteria

1. Volunteers who meet the diagnostic criteria for chronic low back pain;
2. Age between 18 and 60 years old, gender unrestricted, capable of actively participating in treatment;
3. First-time diagnosed patients or those who have not previously received surgical or standard conservative treatments; participants must sign an informed consent form, comply with its terms, and voluntarily agree to take part in this study.

**Exclusion criteria:**

1. Pathogenic factors that contribute to low back pain (symptoms of spinal cord compression, rheumatoid arthritis) are excluded from the diagnostic criteria;
2. Patients with severe cardiovascular and cerebrovascular diseases, metabolic disorders, and mental illnesses;
3. Individuals who have undergone regular physical therapy or other rehabilitation therapies within the past three months.

### 2.2 Aims and hypotheses

The primary objective of this study is to elucidate the therapeutic efficacy of WQXs when integrated with passive recovery and vigorous exercise regimens. Investigating the impact of this combination on lumbar mobility, pain, and quality of life to determine whether the combination of MT and WQXs may alleviate persistent lower back pain. It is hypothesized that the integration of these two modalities would lead to improved quality of life, less discomfort, and increased mobility as compared to either modality applied in isolation.

### 2.3 Participants & recruitment

Enrollment in this study begins on September 1 5, 2023 and will end upon completion of the required subject enrollment. A plan and methodology for subject engagement was established to help subjects understand the purpose and plan of the trial, which may include the preparation of informed consent forms, collection of necessary data, and visual aids such as videos and pictures. During the recruitment process, the advantages and disadvantages of the experimental treatment will be explained in detail, as well as the safety measures that will be taken during the trial, and the implementation of safety protocols will be guaranteed. In addition, wider publicity will be provided through the distribution of recruitment posters in the community and the use of the Internet. The study team will initially assess the eligibility of each subject based on the inclusion/exclusion criteria, followed by relevant examinations. Subsequently, upon the formal signing of the informed consent form, the participants’ adherence to the inclusion and exclusion criteria will be definitively confirmed based on the outcomes of these assessments.

### 2.4 Randomization

Grouping will be done using a computer-generated table of randomized numbers, Random numbers will be assigned by an independent person who will not participate in this study. Random numbers will be placed in opaque envelopes by an independent person who is not involved in this trial to achieve allocation concealment.

### 2.5 Interventions

#### 2.5.1 WQXs

The movements of tiger play (tiger lift, tiger pounce), deer play (necked deer, necked deer) and bear play (bear shake, bear movement) are selected as the basis for the exercise routine design.

**Figure 2:**
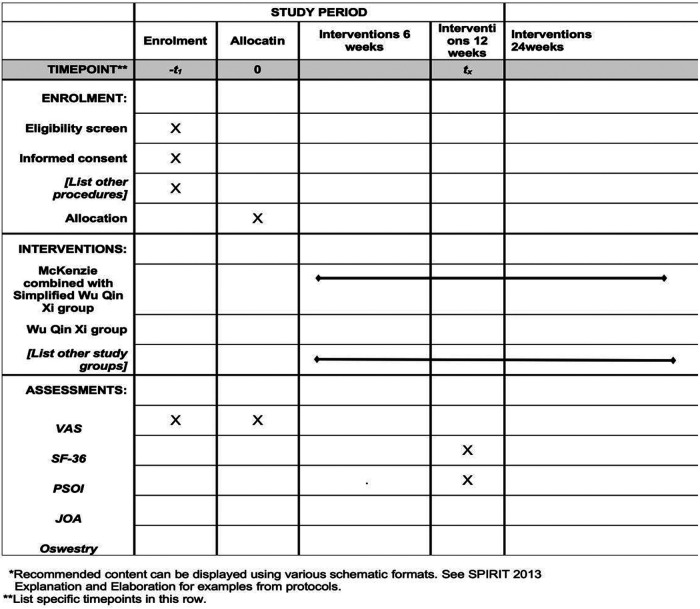
Participant timeline and assessments.

The initial phase consists of a 5-min preparatory warm-up session, followed by a 20-min simplified WQXs exercise targeting the core muscles of the low back and abdomen. Subsequently, the third stage involved a 5-min relaxation training component within the simplified WQXs regimen. In the simplified WQXs training for the core muscles of the lumbar and abdominal regions, a standing and moving position is used, Perform 3 sets of each movement, with 15 repetitions per set, maintaining each repetition for 5 s, and allow a 30 s rest interval between sets. A course of 5 sessions/week, combined with health education and a 2-day break between sessions, The study observation period spanned 24 weeks, comprising a 1-week intervention phase followed by follow-up assessments at weeks 6, 12, 18, and 24 (Figure 2).

#### 2.5.2 MT

The MT method of treatment (application of prone stretching, stretching and relaxation, rotation and relaxation in the stretching position, and continuous rotation in the flexion position) is performed. The method (based on treatment techniques such as prone extension, extension release, extension rotation release, and continuous rotation in flexion) is performed for 10 min, Perform 3 sets of each movement, with 15 repetitions per set, maintaining each repetition for 5 s, and allow a 30 s rest interval between sets. It is advisable to ensure that the interval between each action does not exceed 30 min. 40 min of exercise treatment and health lectures are given to the patients to enhance their compliance. A course of 5 sessions/week, combined with health education and a 2-day break between sessions.

#### 2.5.3 Health education

Patients who participate in the project will be told about the background and purpose of the study of MT and WQXs and the principles and functions of MT and WQXs and emphasize the matters that need to be paid attention to in the process of exercise, to help the patients to find the appropriate intensity, frequency and time, and to help the patients to promote the recovery of lumbar spine function safely and effectively through the exercise.

### 2.6 Outcome measures

During the cycle, participants will undergo an initial assessment at baseline within one week of enrollment, followed by follow-up evaluations at four time points: 0 weeks, 6 weeks, 12 weeks, and 24 weeks. The primary outcome is lumbar lordosis mobility. Secondary outcomes include surface EMG, isometrics muscle strength, the Depression Self-Rating Scale, theVAS score, score of the JOA Lower Back Pain Rating Scale, score of the Oswestry Lumbar Functional Index Questionnaire, score of the SF-36 Healthy Quality of Life Scale, and score of the Pittsburgh Sleep Quality Inventory (PSOI).

### 2.7 Facilitator selection, training and supervision

The following tight controls are placed on the quality of each stage of the study's implementation to guarantee the trial's safety, rigor, and scientific validity:

1. Patient inclusion: Patients are screened in compliance with the study's diagnostic, inclusion, and exclusion criteria.
2. Sample size determination, group allocation, randomization, blinding procedures, and statistical methods are established based on a comprehensive review of relevant literature.
3. Before the pilot study is carried out, the study's personnel receive rigorous training.
4. WQXs train teachers perform WQXs imagery training and provide rich and efficient WQXs demonstrations to guarantee the quality of the motions. Primary therapists conduct comprehensive rehabilitation training and assessments.
5. Bedside physicians and rehabilitation therapists work collaboratively to actively engage participants, clearly explaining the purpose and procedures of the motor imagery intervention until full implementation is achieved.

### 2.8 Patient and public involvement

This preliminary investigation will enroll a diverse group of individuals with chronic low back pain, recruited from the Hongkou and Yangpu districts in Shanghai. All interventions provided for these participants will take place in their respective local communities. The trial will be coordinated and supervised by the Fourth People's Hospital of Tongji University, based in Shanghai, which will also be responsible for managing the trial data.

### 2.9 Blinding

Random allocation will be assigned by an independent person who will not involved in the trial. As this study is an exercise intervention, it will be difficult to conceal the grouping from the therapists and subjects, but it will be possible to blind the outcome measures. A new assessor will be assigned to replace the assessor familiar with the allocation in order to realize single blinding.

### 2.10 Sample size

In this study, the formula for calculating the sample size for the two-sample test of variance was used, with lumbar anterior flexion range of motion as the main effector, and referring to the literature report and the result of the pre-test, the range of motion of lumbar anterior flexion in the two treatment groups was 46.20 ± 5.25 and 42.59 ± 5.14 (26), respectively, which was calculated to require 33 patients in each group. Accounting for an anticipated 10% dropout rate, the total sample size needed is 74 participants.

### 2.11 Statistical analysis plan

According to the Case report form (CRF) table, after each measurement, the data are double-entered into Stata software within one week, and the data are processed and analyzed using SPSS26.0 statistical software. Measurement data are expressed as mean ± standard deviation, and the *t*-test is used if the data meet the normal distribution, and the rank-sum test is used if the data do not meet the normal distribution and the chi-square test was used for comparison of count data. *α* = 0.05 was used as the test level for significant differences.

If there are intolerable or serious adverse reactions during exercise, the trial will be discontinued; however, those who have participated in more than 1 cycle of exercise should be counted for efficacy. Volunteers who are unwilling to continue the clinical trial during the trial and take the initiative to ask the investigator to discontinue the trial could discontinue the trial. In case of discontinuation, more than 1/2 of the data are analyzed and less than 1/2 are excluded.

If imbalances exist between the two groups at baseline regarding variables such as gender, occupation, and physical activity, Analysis of Covariance (ANCOVA) with covariate adjustment will be employed as a sensitivity analysis。

Recognizing that age may influence musculoskeletal elasticity, the speed of rehabilitation response, and compliance, we performed a stratified analysis by age (e.g., <40 years vs. ≥40 years) in addition to conventional analyses, to examine whether there was a significant interaction effect of the intervention across different age groups.

## 3 Ethics and dissemination

### 3.1 Ethics

This project was approved by the Research Ethics Committee of the Fourth People's Hospital of Shanghai Tongji University (No. 2022115-001). All experimental studies were conducted in accordance with the regulations of the Research Ethics Committee of the Fourth People's Hospital affiliated to Tongji University

### 3.2 Data management and trial monitoring

#### 3.2.1 Data collection

Before the initiation of data management for clinical trials, it is imperative to formulate a plan based on the unique characteristics of the research endeavor. The development of a (CRF), design, and completion of this document, as well as its subsequent submission, fall under the purview of the sponsor, commissioned by the Clinical Research Organization the investigators, data management specialists, and statisticians. The design of the database should be anchored in the CRF of the project, with careful attention paid to the receipt and entry of test data. To ensure double-entry accuracy, verifiable measures must be implemented to verify data integrity, including randomization, enrollment, exclusion criteria, time windows, logic, and extreme values. Additionally, challenges from clinical inspectors must be managed appropriately through signed written responses to their queries.

Data challenge forms may also be issued by clinical inspectors to both investigators and researchers. These forms require signed written answers to questions, indicating compliance with regulatory standards. Management of data challenge forms involves responding to these queries from clinical investigators and researchers while maintaining the confidentiality of patient information.

#### 3.2.2 Quality control

During the execution of the study, strict quality control measures are implemented at every stage to guarantee scientific rigor, safety, and accuracy. These stages include patient inclusion according to diagnostic criteria, study inclusion/exclusion criteria, determination of sample size, randomization, single-blind methodology, statistical analysis methods, and more. Before conducting the pilot study, personnel involved in the study receive comprehensive training. All measurements were independently performed by two standardized assessors, and the mean values were adopted. Comprehensive rehabilitation training and assessment are conducted by primary therapists, while animal play demonstrations and imagery training are performed by trained instructors using Five Animal Play methodologies. This ensures that the quality of movement and training content is robust and effective.

From patient inclusion until the implementation of the intervention, hospitalized physicians and rehabilitation therapists collaborate closely with patients, patiently explaining the role of exercise imagery intervention in a clear and understandable manner. This approach fully motivates patients to actively participate in treatment programs, ultimately contributing to the success of the research endeavor.

#### 3.2.3 Publications

Communicating with the outside world through public publications.

## 4 Results

Following ethical guidelines, the present investigation aims to reduce patients’ reliance on drugs, particularly those used for subject-reported symptom control, rather than advocating for changes to patients’ pharmaceutical regimens. The research team will document any changes in the use of medications during a brief time, concentrating only on medication adjustments related to the treatment of low back pain. The objective is to evaluate the therapeutic efficacy of a combined exercise prescription in the treatment of chronic low back pain.

## 5 Discussion

Low back pain (LBP) is a prevalent musculoskeletal condition affecting approximately 70% of the global population at some point in their lives, often leading to limb dysfunction and reduced work capacity (27). The aim of this study is to combine MT technique of the modern exercise rehabilitation with simplified WQXs of the traditional exercise rehabilitation to observe the effects on chronic low back pain.

Patients with low back pain often exhibit decreased lumbar spine mobility, which may be associated with increased paraspinal muscle activity (28–30). Although the assessment of lumbar spinal mobility entails a degree of reliance on the evaluator, the impact of subjective factors was minimized by blinding the assessors to group allocation. The BIODEX III isokinetic multi-joint testing system will be employed to determine peak force distance, total work, and other muscle mechanical performance indexes of the core muscles of the waist and abdomen. In addition, surface electromyography (sEMG) will be employed to capture neuromuscular activation patterns of key core muscles, including the multifidus, transversus abdominis, rectus abdominis, and external abdominal oblique muscles. Surface EMG allows for the measurement of muscle electrical signals after activity and response to muscle coordination (31). To measure the impact of the intervention, a scale is utilized to evaluate the patients’ capacity for daily living as well as their physical function (32). Comparison of changes in the above metrics between the two groups may help explore the potential mechanisms of the changes in mechanical properties of the lumbar and abdominal core muscles in chronic low back pain.

Core strength is strongly linked to low back pain (33) and core stability is essential for proper load balance within the pelvis, spine, and kinetic chain. Core stability exercise (CSE) is an exercise treatment option for low back pain (34). Salik Sengul (35) found that CSE improved pain, endurance, and control in patients with chronic low back pain. Blodt (36) concluded that qigong training was more effective than exercise therapy in relieving pain in patients with chronic low back pain. Liu (37) reported that chronic nonspecific pain in patients over 50 years of age who underwent Chen-style taijiquan training had better pain relief than exercise therapy. It is shown that MT did not increase trunk muscle thickness and did not increase core strength in the trunk (38–40).The Chinese medical notion of “preventing a change in the event of illness” is comparable to the principles of MT, self-treatment, and active prevention. However, the MT lacks advantages and potential dangers in regulating emotions and cardiovascular conditions, while the simplified WQXs is an active training to adjust the physical form and mental state by imitating the movements of animals (41).

Therefore, it is very important to combine the MT technique concept with the exercise routines of the simplified WQXs to form a set of more ideal, superior exercise therapies for treating patients with chronic low back pain. But the research direction of this topic combines the concepts of traditional exercise rehabilitation (simplified WQXs) and modern exercise rehabilitation techniques (MT) to formulate an easier and more effective exercise prescription for treating and preventing low back pain while exploring its effects on low back pain and cardiovascular health. This approach aims to improve clinical efficacy while implementing a low-cost, high-efficiency, scientific modern gong method to address a common high morbidity issue and play a positive role. Although this study did not systematically evaluate the participants’ static posture or dynamic postural patterns, the intervention itself incorporated elements of posture correction. This study did not distinguish the independent effects of each intervention factor in the treatment of low back pain. In the future, the sample size should be increased and more subgroups included to investigate the independent effects of each intervention factor. Future research should integrate posture assessment tools (e.g., photogrammetry or motion capture systems) to quantify habitual posture and analyze its association with therapeutic efficacy. Subsequent studies will adopt a multicenter, stratified randomized design to more precisely control for confounding variables.

## References

[1] Maher C Underwood M Buchbinder R. Non-specific low back pain. Lancet. (2017) 389(10070):736–47. 10.1016/S0140-6736(16)30970-9 27745712

[2] Urits I Burshtein A Sharma M Testa L Gold PA Orhurhu V Low back pain, a comprehensive review: pathophysiology, diagnosis, and treatment. Curr Pain Headache Rep. (2019) 23(3):23. 10.1007/s11916-019-0757-1 30854609

[3] Bogduk N. On the definitions and physiology of back pain, referred pain, and radicular pain. Pain. (2009) 147(1-3):17–9. 10.1016/j.pain.2009.08.020 19762151

[4] Airaksinen O Brox JI Cedraschi C Hildebrandt J Klaber-Moffett J Kovacs F Chapter 4. European guidelines for the management of chronic nonspecific low back pain. Eur Spine J. (2006) 15(Suppl 2):S192–300. 10.1007/s00586-006-1072-1 16550448 PMC3454542

[5] Atlas SJ Deyo RA. Evaluating and managing acute low back pain in the primary care setting. J Gen Intern Med. (2001) 16(2):120–31. 10.1111/j.1525-1497.2001.91141.x 11251764 PMC1495170

[6] Heuch I Foss IS. Acute low back usually resolves quickly but persistent low back pain often persists. J Physiother. (2013) 59(2):127. 10.1016/S1836-9553(13)70166-8 23663799

[7] Balagué F Mannion AF Pellisé F Cedraschi C. Non-specific low back pain. Lancet. (2012) 379(9814):482–91. 10.1016/S0140-6736(11)60610-7 21982256

[8] Crow WT Willis DR. Estimating cost of care for patients with acute low back pain: a retrospective review of patient records. J Am Osteopath Assoc. (2009) 109(4):229–33. 10.7556/jaoa.2009.109.4.229 19369510

[9] Miller P Kendrick D Bentley E Fielding K. Cost-effectiveness of lumbar spine radiography in primary care patients with low back pain. Spine. (2002) 27(20):2291–7. 10.1097/00007632-200210150-00021 12394910

[10] Balki S Göktas HE. Short-Term effects of the kinesio taping® on early postoperative hip muscle weakness in male patients with hamstring autograft or allograft anterior cruciate ligament reconstruction. J Sport Rehabil. (2019) 28(4):311–7. 10.1123/jsr.2017-0219 29252113

[11] Witenko C Moorman-Li R Motycka C Duane K Hincapie-Castillo J Leonard P Considerations for the appropriate use of skeletal muscle relaxants for the management of acute low back pain. P & T. (2014) 39(6):427–35. 25050056 PMC4103716

[12] Pincus T Vlaeyen JW Kendall NA Von Korff MR Kalauokalani DA Reis S. Cognitive-behavioral therapy and psychosocial factors in low back pain: directions for the future. Spine. (2002) 27(5):E133–8. 10.1097/00007632-200203010-00020 11880850

[13] National Guideline Centre (UK). Low Back Pain and Sciatica in Over 16s: Assessment and Management NICE Guideline. London: National Institute for Health and Care (2016). 27929617

[14] Bishop MD Torres-Cueco R Gay CW Lluch-Girbés E Beneciuk JM Bialosky JE. What effect can manual therapy have on a patient's Pain experience? Pain Manag. (2015) 5(6):455–64. 10.2217/pmt.15.39 26401979 PMC4976880

[15] Bernal-Utrera C Gonzalez-Gerez JJ Anarte-Lazo E Rodriguez-Blanco C. Manual therapy versus therapeutic exercise in non-specific chronic neck pain: a randomized controlled trial. Trials. (2020) 21(1):682. 10.1186/s13063-020-04610-w 32723399 PMC7385865

[16] Chou R Huffman LH; American Pain Society, & American College of Physicians. Nonpharmacologic therapies for acute and chronic low back pain: a review of the evidence for an American pain society/American college of physicians clinical practice guideline. Ann Intern Med. (2007) 147(7):492–504. 10.7326/0003-4819-147-7-200710020-00007 17909210

[17] Machado LA Maher CG Herbert RD Clare H McAuley JH. The effectiveness of the McKenzie method in addition to first-line care for acute low back pain: a randomized controlled trial. BMC Med. (2010) 8:10. 10.1186/1741-7015-8-10 20102596 PMC2842230

[18] Ng BH Tsang HW. Psychophysiological outcomes of health qigong for chronic conditions: a systematic review. Psychophysiology. (2009) 46(2):257–69. 10.1111/j.1469-8986.2008.00763.x 19170945

[19] O’Sullivan PB Phyty GD Twomey LT Allison GT. Evaluation of specific stabilizing exercise in the treatment of chronic low back pain with radiologic diagnosis of spondylolysis or spondylolisthesis. Spine. (1997) 22(24):2959–67. 10.1097/00007632-199712150-00020 9431633

[20] Hides JA Jull GA Richardson CA. Long-term effects of specific stabilizing exercises for first-episode low back pain. Spine. (2001) 26(11):E243–8. 10.1097/00007632-200106010-00004 11389408

[21] Norris C. Spinal stabilisation.: part 1. Active lumbar stabilisation concepts. Physiotherapy. (1995) 81:61–4. 10.1016/S0031-9406(05)67046-0

[22] Norris C. Spinal stabilisation.: part 3. Stablisation mechanisms of the lumbar spine. Physiotherapy. (1995) 81:72–9. 10.1016/S0031-9406(05)67048-4

[23] Macdonald DA Dawson AP Hodges PW. Behavior of the lumbar multifidus during lower extremity movements in people with recurrent low back pain during symptom remission. J Orthop Sports Phys Ther . (2011) 41(3):155–64. 10.2519/jospt.2011.3410 21212497

[24] Hides J Stanton W Mendis MD Sexton M. The relationship of transversus abdominis and lumbar multifidus clinical muscle tests in patients with chronic low back pain. Man Ther. (2011) 16(6):573–7. 10.1016/j.math.2011.05.007 21641268

[25] Brenner AK Gill NW Buscema CJ Kiesel K. Improved activation of lumbar multifidus following spinal manipulation: a case report applying rehabilitative ultrasound imaging. J Orthop Sports Phys Ther . (2007) 37(10):613–9. 10.2519/jospt.2007.2470 17970408

[26] Xuyong X Huizhen Z Hongbin Y. Application of McKenzie therapy combined with suspension core training in elderly patients with lumbar intervertebral disc herniation China Med Innov. (2023) 20(25):168–72. 10.3969/j.issn.1674-4985.2023.25.039

[27] Andersson GB. Epidemiological features of chronic low-back pain. Lancet. (1999) 354(9178):581–5. 10.1016/S0140-6736(99)01312-4 10470716

[28] Chen WJ Chiou WK Lee YH Lee MY Chen ML. Myo-electric behavior of the trunk muscles during static load holding in healthy subjects and low back pain patients. Clin Biomech. (1998) 13(Suppl 1):S9–15. 10.1016/s0268-0033(98)80133-2 11430785

[29] Larivière C Gagnon D Loisel P. The comparison of trunk muscles EMG activation between subjects with and without chronic low back pain during flexion-extension and lateral bending tasks. J Electromyogr Kinesiol. (2000) 10(2):79–91. 10.1016/s1050-6411(99)00027-9 10699556

[30] Lehman GJ. Clinical considerations in the use of surface electromyography: three experimental studies. J Manipulative Physiol Ther. (2002) 25(5):293–9. 10.1067/mmt.2002.124423 12072849

[31] Krekoukias G Petty NJ Cheek L. Comparison of surface electromyographic activity of erector spinae before and after the application of central posteroanterior mobilisation on the lumbar spine. J Electromyogr Kinesiol. (2009) 19(1):39–45. 10.1016/j.jelekin.2007.06.020 17888680

[32] Lins L Carvalho FM. SF-36 total score as a single measure of health-related quality of life: scoping review. SAGE Open Med. (2016) 4:2050312116671725. 10.1177/2050312116671725 27757230 PMC5052926

[33] Fernández-Rodríguez R Álvarez-Bueno C Cavero-Redondo I Torres-Costoso A Pozuelo-Carrascosa DP Reina-Gutiérrez S Best exercise options for reducing pain and disability in adults with chronic low back pain: pilates, strength, core-based, and mind-body. A network meta-analysis. J Orthop Sports Phys Ther . (2022) 52(8):505–21. 10.2519/jospt.2022.10671 35722759

[34] Kim B Yim J. Core stability and hip exercises improve physical function and activity in patients with non-specific low back pain: a randomized controlled trial. Tohoku J Exp Med. (2020) 251(3):193–206. 10.1620/tjem.251.193 32669487

[35] Salik Sengul Y Yilmaz A Kirmizi M Kahraman T Kalemci O. Effects of stabilization exercises on disability, pain, and core stability in patients with non-specific low back pain: a randomized controlled trial. Work. (2021) 70(1):99–107. 10.3233/WOR-213557 34487008

[36] Blödt S Pach D Kaster T Lüdtke R Icke K Reisshauer A Qigong versus exercise therapy for chronic low back pain in adults–a randomized controlled non-inferiority trial. Eur J Pain. (2015) 19(1):123–31. 10.1002/ejp.529 24902673

[37] Liu J Yeung A Xiao T Tian X Kong Z Zou L Chen-Style tai chi for individuals (aged 50 years old or above) with chronic non-specific low back pain: a randomized controlled trial. Int J Environ Res Public Health. (2019) 16(3):517. 10.3390/ijerph16030517 30759778 PMC6388249

[38] Halliday MH Pappas E Hancock MJ Clare HA Pinto RZ Robertson G A randomized controlled trial comparing the McKenzie method to motor control exercises in people with chronic low back pain and a directional preference. J Orthop Sports Phys Ther. (2016) 46(7):514–22. 10.2519/jospt.2016.6379 27170524

[39] Miller ER Schenk RJ Karnes JL Rousselle JG. A comparison of the McKenzie approach to a specific spine stabilization program for chronic low back pain. J Man Manip Ther. (2005) 13(2):103–12. 10.1179/106698105790824996

[40] Hosseinifar M Akbari M Behtash H Amiri M Sarrafzadeh J. The effects of stabilization and McKenzie exercises on transverse abdominis and multifidus muscle thickness, pain, and disability: a randomized controlled trial in nonspecific chronic low back pain. J Phys Ther Sci. (2013) 25(12):1541–5. 10.1589/jpts.25.1541 24409016 PMC3885835

[41] Lee SH Jeon Y Huang CW Cheon C Ko SG. Qigong and tai chi on human health: an overview of systematic reviews. Am J Chin Med (Gard City N Y). (2022) 50(8):1995–2010. 10.1142/S0192415X22500859 36266755

